# Candidate circulating microRNA biomarkers in dogs with chronic pancreatitis

**DOI:** 10.1111/jvim.17009

**Published:** 2024-02-13

**Authors:** Susan K. Armstrong, Robert W. Hunter, Wilna Oosthyuzen, Maciej Parys, Adam G. Gow, Silke Salavati Schmitz, James W. Dear, Richard J. Mellanby

**Affiliations:** ^1^ School of Veterinary Medicine University of Surrey, Guildford Surrey United Kingdom; ^2^ Edinburgh Kidney, Centre for Cardiovascular Science University of Edinburgh Edinburgh United Kingdom; ^3^ The Royal (Dick) School of Veterinary Studies and the Roslin Institute University of Edinburgh Edinburgh United Kingdom; ^4^ Zoetis UK Ltd Leatherhead United Kingdom; ^5^ Centre for Precision Cell Therapy for the Liver, Lothian Health Board Queens Medical Research Institute Edinburgh United Kingdom; ^6^ Idexx Wetherby United Kingdom

**Keywords:** blood, diagnostics, gastrointestinal, liquid biopsy, transcriptomics

## Abstract

**Background:**

Pancreatitis is an important cause of disease and death in dogs. Available circulating biomarkers are not sufficiently sensitive and specific for a definitive diagnosis.

**Hypothesis:**

Circulating microRNAs would be differentially expressed in dogs with chronic pancreatitis and could have potential as diagnostic biomarkers.

**Animals:**

Healthy controls (n = 19) and dogs with naturally occurring pancreatitis (n = 17).

**Methods:**

A retrospective case‐control study. Dogs with pancreatitis were included if they satisfied diagnostic criteria for pancreatitis as adjudicated by 3 experts. MicroRNA was extracted from stored serum samples and sequenced. Reads were mapped to mature microRNA sequences in the canine, mouse, and human genomes. Differentially expressed microRNAs were identified and the potential mechanistic relevance explored using Qiagen Ingenuity Pathway Analysis (IPA).

**Results:**

Reads mapping to 196 mature microRNA sequences were detected. Eight circulating microRNAs were significantly differentially expressed in dogs with pancreatitis (≥2‐fold change and false discovery rate <0.05). Four of these mapped to the canine genome (cfa‐miR‐221, cfa‐miR‐222, cfa‐miR‐23a, and cfa‐miR‐205). Three mapped to the murine genome (mmu‐miR‐484, mmu‐miR‐6240, mmu‐miR‐101a‐3p) and 1 to the human genome (hsa‐miR‐1290). Expression in dogs with pancreatitis was higher for 7 microRNAs and lower for mmu‐miR‐101a‐3p. Qiagen IPA demonstrated a number of the differently expressed microRNAs are involved in a common pancreatic inflammatory pathway.

**Conclusions:**

The significantly differentially expressed microRNAs represent promising candidates for further validation as diagnostic biomarkers for canine pancreatitis.

**Abbreviations**
cPL: canine pancreas‐specific lipase immunoreactivity
CPM: counts per million reads mapped
FDR: false discovery rate
HfSA: Hospital for Small Animals
ICAM‐1: intracellular adhesion molecule‐1
ICOS: inducible co‐stimulator
IPA: ingenuity pathway analysis
IQR: inter‐quartile range
M: million
miR: microRNA
PTSG2: prostaglandin‐2
UMI: unique molecular identifier

## 1 INTRODUCTION

Pancreatitis in dogs is a highly debilitating and painful disease. It is estimated that over 30% of dogs will develop some degree of pancreatitis in their lifetime, with prevalence of chronic pancreatitis as high as 34% (51/151) in 1 study of dogs at post‐mortem.^1^, ^2^ Chronic pancreatitis in dogs is associated with refractory pain and reduced quality of life, precipitating progressive life‐limiting impairment of pancreatic function.^3^, ^4^, ^5^, ^6^ There are currently few substantive pathophysiological studies in dogs with naturally occurring pancreatitis, so disease insight is often extrapolated from humans and animal models.^7^, ^8^, ^9^, ^10^

Antemortem diagnosis of pancreatitis in dogs is challenging, relying heavily on clinical history and a combination of diagnostic tests, all of which have limitations.^3^, ^11^ Histopathology is generally considered the reference diagnostic standard, but is imperfect.^11^, ^12^, ^13^ Improvements are reported in evaluating the pancreas through noninvasive imaging techniques such as ultrasonography and computed tomography, but a number of factors, including operator experience, can highly influence their diagnostic utility.^4^, ^14^, ^15^, ^16^, ^17^, ^18^, ^19^

All currently available circulating biomarkers for pancreatitis diagnosis have suboptimal testing accuracy depending on severity of disease, highlighting the need for better biomarker candidates.^11^, ^14^, ^20^, ^21^, ^22^, ^23^, ^24^ The most frequently used assay is canine pancreas‐specific lipase immunoreactivity (cPL), available as both a quantitative (Spec cPL) assay and a semi‐quantitative point‐of‐care (SNAP) cPL test.^11^, ^14^, ^21^ Spec cPL is generally considered the most sensitive and specific test for pancreatitis in dogs, but requires analysis in a reference laboratory.^21^, ^24^ In 1 study, dogs with concurrent acute and chronic pancreatitis, Spec cPL sensitivity was moderate (71%) and specificity was variable depending on the cPL cut‐off used (86‐100%).^24^ In 8 dogs with chronic pancreatitis 1,2‐*o*‐dilauryl‐rac‐glycero‐3‐glutaric acid‐(6′‐methylresorufin) ester lipase specificity was 100%, but sensitivity was only 57%.^25^, ^26^ MicroRNAs are emerging as sensitive and specific markers of pancreatitis in humans.^27^, ^28^, ^29^ We hypothesized that small RNA‐sequencing of serum from dogs with pancreatitis and undertaking post‐sequencing analysis would lead to identification of targeted microRNAs that could be further interrogated as diagnostic biomarkers of naturally occurring chronic pancreatitis in dogs.

## 2 MATERIALS AND METHODS

A retrospective study, for which all dogs were enrolled for at the Royal (Dick) School of Veterinary Studies, University of Edinburgh, United Kingdom. Dogs presented to the Hospital for Small Animals (HfSA) as referral cases or for routine appointments. They were only considered for the study if a blood sample was undertaken as part of their routine clinical investigation and residual serum was stored in the HfSA biobank. Dogs were initially selected (n = 24) by Veterinary nomenclature retrospective search for a diagnosis of pancreatitis. Dogs allocated a diagnosis of pancreatitis then retrospectively had their pancreatitis grade scored by 3 blinded HfSA senior boarded medicine clinicians according to the system described by McCord et al. in the absence of a validated chronic pancreatitis score.^21^ In brief, dogs were stratified by a likelihood score for pancreatitis of 0 to 4. For this, 0: definitely not pancreatitis, 1: probably not pancreatitis, 2; possibly pancreatitis, 3: probably pancreatitis, 4: definitely pancreatitis. Only dogs graded 2 and above were included in the study (n = 17). Three dogs were graded as probably having pancreatitis (grade 3) and 14 dogs for possibly having pancreatitis (grade 2). Each dog was independently scored after reviewing the history, results of routine hematology, serum biochemistry, SNAP cPL test, cPL assay, abdominal ultrasound, further diagnostic procedures, including histopathology, final outcome, and final diagnosis as available. The panel evaluated the dogs together, but assigned a score independently. Blood samples from healthy dogs exhibiting no signs of systemic disease were included in the study as controls. These samples were already present in the existing biobank. Attempt was made to match control samples as far as possible to dogs with pancreatitis for age, sex, breed, and time in storage. This study was approved by the University of Edinburgh Veterinary Ethics Research Committee (approval number: 117.22).

### 2.1 Sample storage and RNA isolation

Serum was placed in −20°C within 12 hours of sample collection. The serum was then transferred within 24 hours to long‐term storage at −80°C.^30^, ^31^ Mean blood sample storage time was 1653 days (range, 0‐2144 days) for the control group and 1641 days (range, 0‐2316 days) for the pancreatitis group (*P* = .83). MicroRNA was extracted in 2 batches using a miRNeasy Serum/Plasma kit (Qiagen, The Netherlands) following the manufacturer's guidelines and as per Vliegenthart et al. and Oosthuyzen et al.^32^, ^33^ Briefly, total RNA was extracted from 50 μL of serum diluted in 150 μL nuclease‐free water. RNA was extracted using lysis reagent (1000 μL) and chloroform (200 μL). The RNA was purified on a RNeasy miniElute spin column and eluted in 15 μL RNase‐free water and stored at −80°C. Extraction efficiency was assessed by adding 6 × 10^9^ copies/μL of synthetic *Caenorhabditis elegans* miR‐39 spike‐in control (Norgen Biotek, Canada) after the addition of lysis reagent.

### 2.2 Library preparation

Thirty‐six sequencing libraries were prepared from microRNA samples using the QIAseq miRNA library kit according to the provided protocol for RNA isolated from serum, 5 μL of each RNA sample was used as input to library construction. In this protocol, a unique molecular identifier (UMI) was introduced during complementary DNA synthesis, to facilitate read counting in the sequencing dataset. Libraries were purified using QIASeq miRNA NGS Beads (QMN beads). Purified libraries were then amplified for 22 cycles of PCR. Amplified libraries were also purified and size selected with QMN beads to enrich for library fragments from microRNA.

### 2.3 Library quality control

Libraries were quantified by fluorometry using the Qubit dsDNA High Sensitivity (HS) assay and assessed for quality and fragment size using the Agilent Bioanalyser with the DNA HS Kit. Libraries were purified by electrophoresis on 3% agarose gels, to remove fragments of inappropriate sizes, such as adapter‐dimers. The final library was then quantified by fluorometry and assessed on the Agilent Bioanalyser to ensure removal of adapter‐dimers.

### 2.4 Sequencing

Sequencing was performed on the NextSeq 2000 platform (Illumina Inc, USA) using NextSeq 1000/2000 P2 Reagents (100 Cycles) v3. PhiX Control v3 library (Illumina Inc, USA) was spiked in at a concentration of 1% to enable troubleshooting in the event of run failure. Sequencing was single‐end 1 × 75.

### 2.5 Data analysis

Data were summarized as median and range for age of study subjects. Sample time in storage analysis was calculated by non‐parametric Mann‐Whitney *U* test. Nominal *P* values equal to or less than .05 were considered significant. Statistical analyses were performed using Graphpad Prism (GraphPad Software, La Jolla, California, v9). Small RNA‐sequencing reads were trimmed using cutadapt, retaining reads between 18 and 30 nucleotides. Reads sharing a UMI were identified with umi_tools and de‐duplified using the “unique” method. Reads were mapped first to the spike‐in sequence (cel‐miR‐39) then unmapped reads were mapped sequentially to mature microRNA sequences in the dog, then human, then mouse genomes (miRBase release 22.1). Mapping was performed using Bowtie, allowing up to 1 nucleotide mismatch within a 32 nucelotide seed region and ignoring reads mapping to more than 1 region.

Differential expression analysis was performed in R (version 4.1.3) using edgeR (v3.36.0). Low‐abundance reads were filtered out and then counts were normalized using the trimmed mean of *M* values method. Differential expression was determined using a 2‐group generalized log‐linear model. Genes were deemed to be differentially expressed if their expression was at least 2‐fold different between the control and pancreatitis groups and false discovery rate (FDR) was less than 5% after adjustment for multiple testing using the Benjamini and Hochberg method.^34^ The heatmap was generated using the pheatmap package (v1.0.12); all other plots were generated using the tidyverse (v1.3.1) and ggplot2 (v3.3.5). The code for this miRNAseq analysis pipeline is publicly accessible at https://zenodo.org/badge/latestdoi/468419301. Further analysis of the significantly differentially expressed microRNAs was conducted by generating networks through the use of QIAGEN Ingenuity Pathway Analysis.^35^

## 3 RESULTS

### 3.1 Canine characteristics

There were samples from 17 dogs with pancreatitis (grade 2 and above) and 19 samples from healthy dogs. Seventeen of the dogs were male (47%) and 19 were female (53%). Ten (9/17) dogs with pancreatitis had no known concomitant disease at time of presentation. Characteristics of the dogs including sex, age, and breed are summarized in Table 1.

**TABLE 1:** Characteristics of dogs included in the study based on allocated group (canine control or canine pancreatitis).

	Healthy control group (n = 19)	Pancreatitis group (n = 17)
Median (IQR) age (years)	6.9 (2.4‐12.6)	8 (2‐13)
Sex (female: male)	9:10	10:7
Breed	Labrador (n = 4), Cocker Spaniel (n = 4), Boxer cross‐breed (n = 2), Golden retriever, Labrador cross‐breed, cross‐breed, West Highland White terrier, Lurcher, Border Terrier	Cocker Spaniels (n = 3), Labrador (n = 2), Cross‐breeds (n = 2), Boxer (n = 2), Miniature Schnauzer (n = 2), West Highland White terrier, Bulldog, Border Terrier, French bulldog, Border Terrier, Beagle, Springer Spaniel
Final diagnosis in pancreatitis group		Pancreatitis (n = 9); pancreatitis and secondary cholangiohepatitis (n = 2); chronic pancreatitis and hepatitis; pancreatitis, atrial standstill, chronic heart failure, pre‐renal azotemia; acute necrotizing pancreatitis, IBD; pancreatitis, cholangiohepatitis, enteritis, esophagitis, leptospirosis; pancreatitis, IBD, UTI; pancreatitis, immune‐mediated Pancytopenia (all n = 1)

Abbreviations: IBD, inflammatory bowel disease; IQR, inter‐quartile range; UTI, urinary tract infection.

### 3.2 Small RNA‐sequencing analysis

**FIGURE 1:**
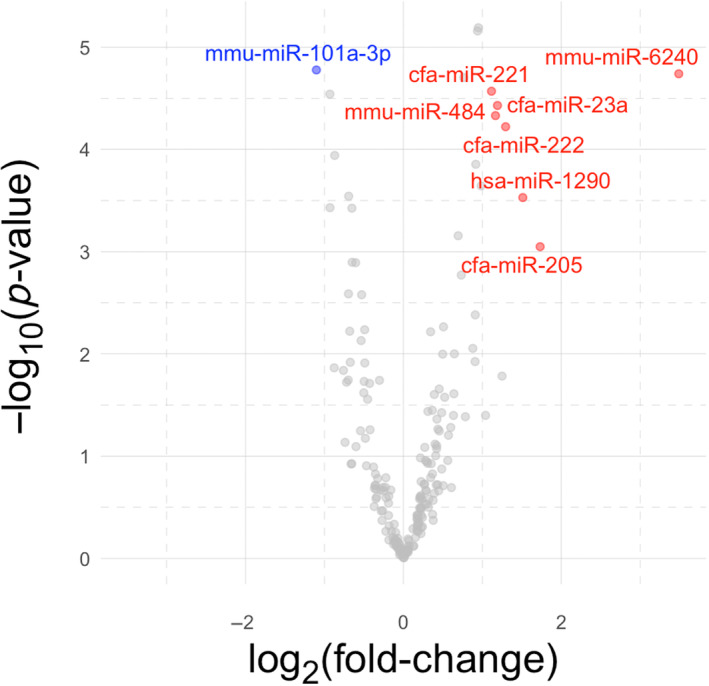
Volcano plot showing differential expression of genes in the pancreatitis group (n = 17), relative to the control group (n = 19). These were mapped to the canine genome (cfa), the murine genome (mmu), and human genome (hsa). Red and blue points show genes deemed to be significantly differentially expressed on the basis of a fold‐change greater than 2 and a false discovery rate less than 5% (blue dots, reduced microRNA differential expression; red dots, increased microRNA differential expression; gray dots, not significantly differentially expressed).

**FIGURE 2:**
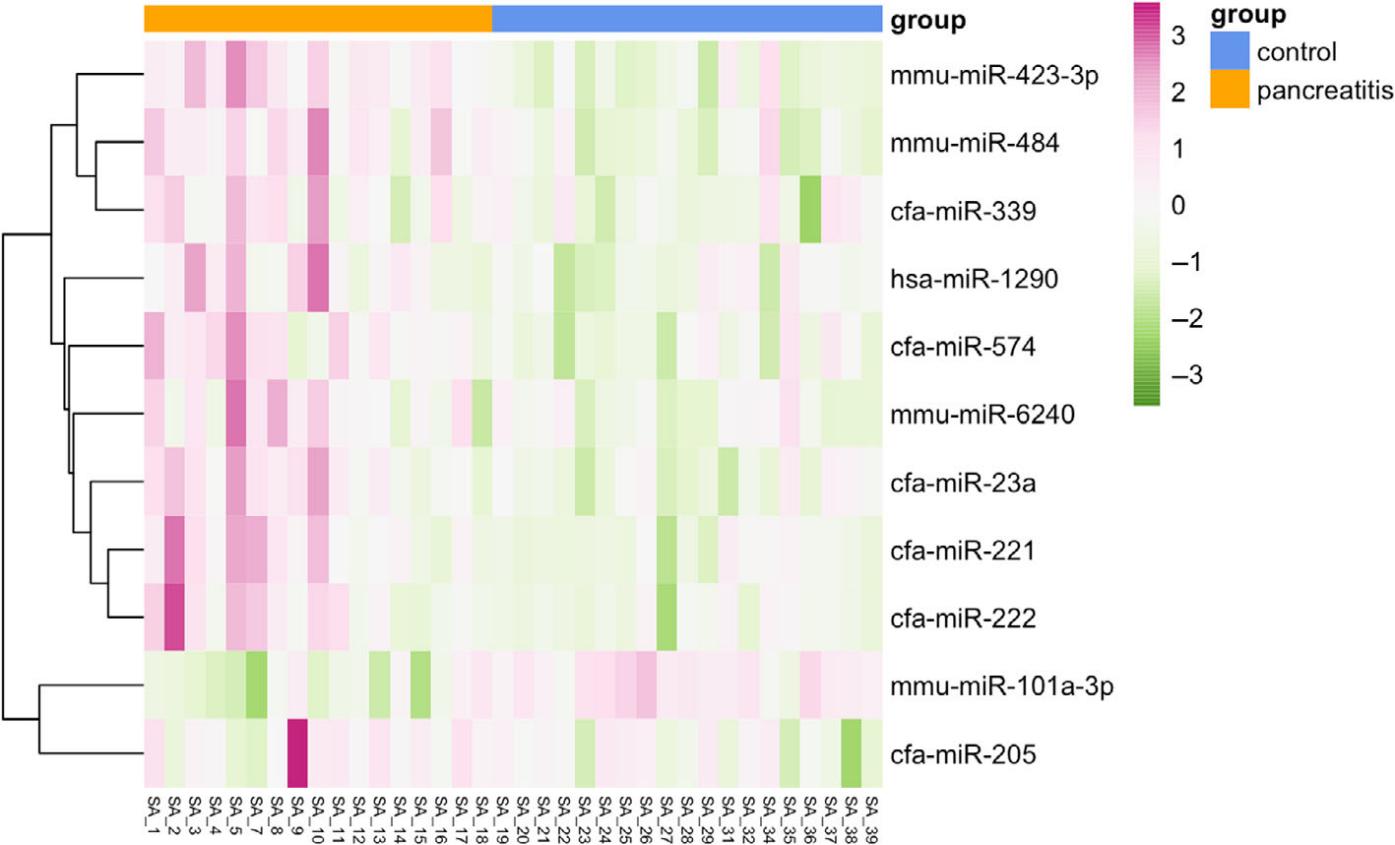
Heatmap of relative expression of the 8 canine microRNAs that were significantly differentially expressed in the pancreatitis group (n = 17) relative to the control group (n = 19). The color scale shows the microRNA expression (pink = high; green = low) within each subject, relative to other subjects for that microRNA (ie, to make within‐row, but not between‐row, comparisons).

**FIGURE 3:**
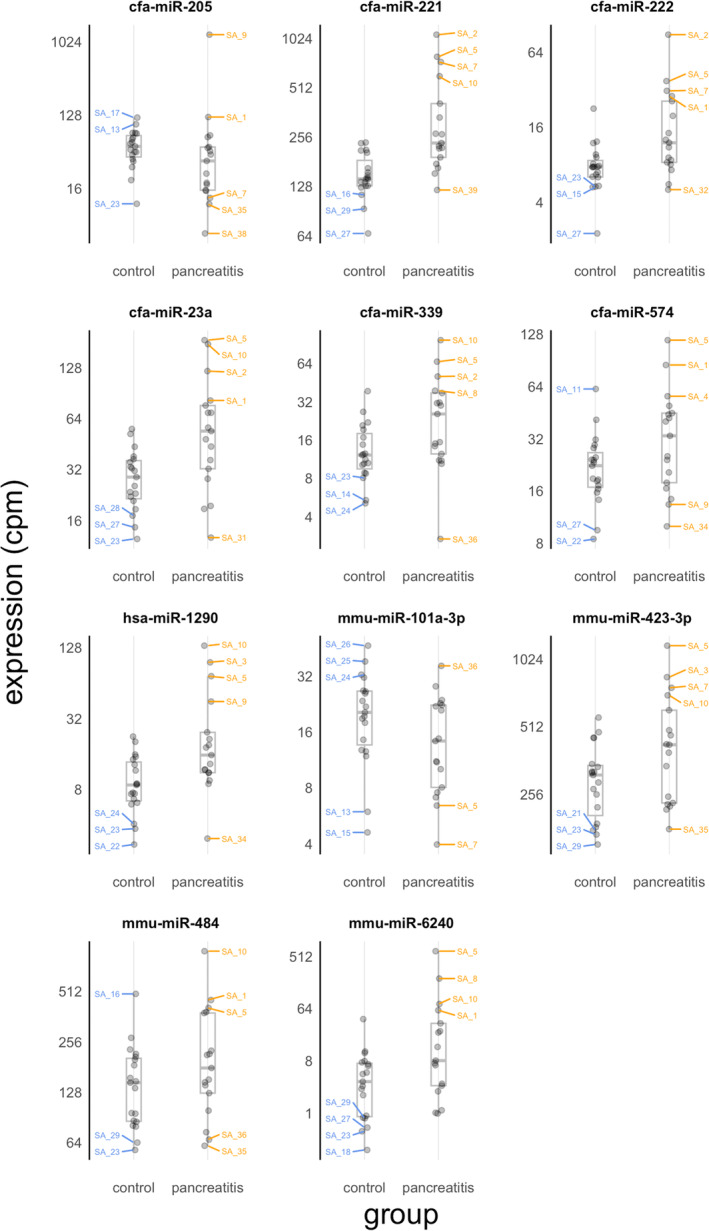
Dot plots showing expression of the 8 microRNAs that were significantly differentially expressed between the 2 groups (n = 17 pancreatitis cases, n = 19 control dogs). The data are shown for individual subjects. The boxplots show group medians (middle line) and upper and lower quartiles (extent of box) on a log2 scale. The whiskers extend from the box to 1.5 times the interquartile range (CPM, counts per million). Labels showing dog identity are included for points that are outside the 10th to 90th centile.

**FIGURE 4:**
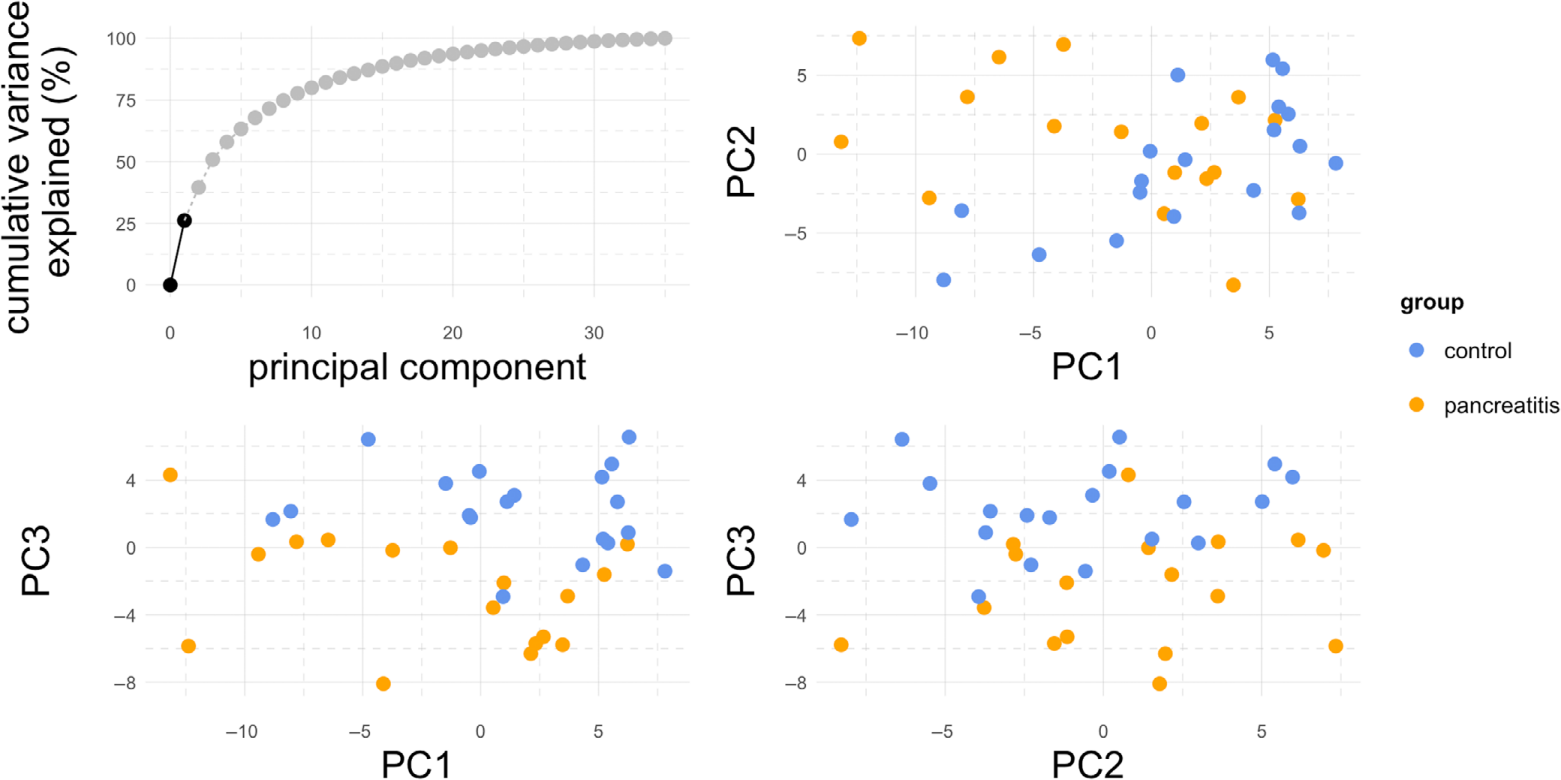
Principal component analysis of small RNA‐sequencing data. The control (n = 19 in blue) and pancreatitis (n = 17 in orange) groups formed distinct (but overlapping) clusters, showing that there were global changes in microRNA expression in pancreatitis. The third principal component, which accounted for 11.2% of the total variance, was most significantly associated with disease status (*P* = 0.0001 by ANOVA).

In the raw sequencing dataset, there were a median of 13.7 million (M) reads per library (inter‐quartile range [IQR], 12.9 M‐14.7 M). After trimming and de‐duplication of UMIs, median library size was 7.3 M reads (6.6 M‐8.0 M) and the reads mapped to 756 unique mature microRNA sequences. After filtering out microRNAs expressed at very low abundance, median library size was 444 000 and reads mapped to 196 mature microRNAs. Of these, 113 had higher expression in the pancreatitis group and 83 had lower expression in the pancreatitis group. Seven microRNAs were significantly differentially upregulated in dogs with pancreatitis; 1 microRNA was downregulated (Table 2; log _2_ [fold‐change] +2, FDR <5%, *P* < .05). Of these 4 mapped to the canine (cfa) genome, 3 to murine (mmu) and 1 to human (hsa) genomes. We present these data as a table (Table 2), Volcano plot (Figure 1), heatmap (Figure 2), and dotplots to show the expression patterns of each differentially‐regulated microRNA (Figure 3). Correlation plots between the microRNAs showed expression was significantly correlated with 2 sets of microRNAs: miR‐222, miR‐221, and miR‐23a; and miR‐23a, miR‐484, and miR‐1290 (Supplementary Figure 1). Principal component analysis was conducted and revealed that the control and pancreatitis groups formed (overlapping) clusters illustrating global changes in dogs diagnosed with pancreatitis (Figure 4). The third principal component, which accounted for 11.2% of the total variance, was most significantly associated with disease status (*P* = .0001 by analysis of variance [ANOVA]).

**TABLE 2:** Analysis of the 8 differentially expressed microRNAs between the canine pancreatitis group (n = 19) and healthy dog group (n = 17).

MicroRNA	Fold‐change	Log_2_ fold‐change	logCPM	*F*‐value	*P*‐value	FDR
**Upregulated**						
mmu‐miR‐6240	11.21	3.49	7.19	23.65	<0.001	<0.001
cfa‐miR‐221	2.17	1.11	10.13	22.46	<0.001	<0.001
cfa‐miR‐23a	2.28	1.19	7.73	21.50	<0.001	<0.001
mmu‐miR‐484	2.24	1.17	9.82	20.83	<0.001	0.001
cfa‐miR‐222	2.45	1.29	6.00	20.11	<0.001	0.001
hsa‐miR‐1290	2.85	1.51	6.52	15.71	<0.001	0.004
cfa‐miR‐205	3.32	1.73	8.56	12.88	<0.001	0.009
**Downregulated**						
mmu‐miR‐101a‐3p	0.47	−1.10	6.49	23.91	<0.001	<0.001

*Note*: Differential expression was determined using a 2‐group generalized log‐linear model. Genes were deemed to be differentially expressed if their expression was at least 2‐fold different between the control and pancreatitis groups and false‐discovery rate was less than 5% after adjustment for multiple testing using the Benjamini and Hochberg method.^47^

Abbreviations: FDR, false discovery rate; logCPM, log_2_‐counts per million.

**FIGURE 5:**
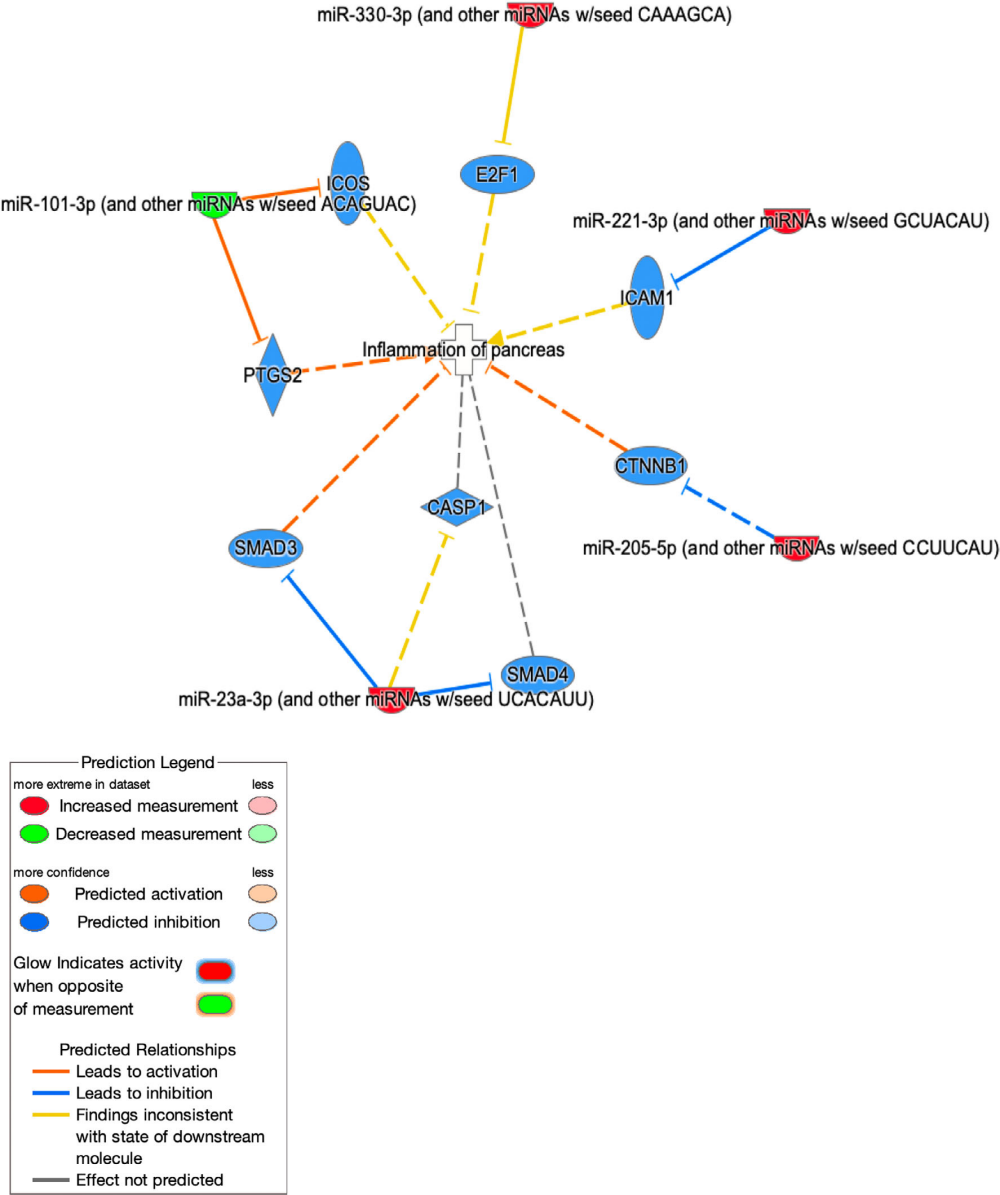
MicroRNA Qiagen IPA network analysis with predicted pathogenic relationship to pancreatitis based on the 8 differentially expressed genes between the control group (n = 19) and pancreatitis group (n = 17). Molecules are represented as nodes, and the biological relationship between 2 nodes is represented as an edge (line). The intensity of the node color indicates the degree of upregulation (red) or downregulation (green). Nodes are displayed using various shapes that represent the functional class of the product. As miR‐211 and miR‐222 are from the same seed sequence Ingenuity Pathway Analysis (IPA) did not differentiate between them.

Using Qiagen Ingenuity Pathway Analysis (IPA) databases, analysis was performed on the network of potential interactions among the set of 8 differentially expressed genes, focusing on molecular pathways involved in inflammation of the pancreas. A Qiagen IPA network/My Pathways is a graphical representation of the molecular relationships between molecules. All edges (relationship between 2 nodes) are supported by at least 1 reference from the literature, from a textbook, or from canonical information stored in the Qiagen Knowledge Base. Human, mouse, and rat orthologs of a gene are stored as separate objects in the Qiagen Knowledge Base, but are represented as a single node in the network. Four of 8 microRNAs (miR‐23a, miR‐101a‐3p, miR‐205, miR‐221) were identified as potentially involved in different (1 or more) pancreatitis‐related pathways (Figure 5).

## 4 DISCUSSION

This study used small RNA‐sequencing to globally assess circulating microRNAs in dogs with naturally occurring chronic pancreatitis. We identified 8 significantly differentially expressed microRNAs. Pancreatic injury induced using a caerulein infusion in 2 dog studies identified microRNAs which could be utilized for pancreatitis diagnosis.^36^, ^37^ Higher levels of miR‐216a, miR‐216b, and miR‐217, miR‐375, and miR‐148a are detected in dogs when pancreatitis is induced.^37^ Of 20 dogs with naturally occurring acute pancreatitis, a significant difference in the serum expression of cfa‐miR‐375 was found between dogs with acute pancreatitis (median: 3.59) and healthy dogs (0.81; *P* < .001), but miR‐216a was not significantly increased in this population.^38^ None of these microRNAs were in the top 20 differentially expressed microRNAs in this study cohort. The difference in microRNA expression profiles between these studies and the current work could be explained by the acute pancreatitis phenotype and the use of the caerulein infusion, which is more a model of acute pancreatitis.^10^ Caerulein infusion can result in highly variable fibrosis and infusion rates vary between studies, which could also contribute to the altered microRNA profiles.^6^ The differences between these studies supports the investigation of naturally occurring pancreatitis in dogs rather than injury models and differentiating between acute and chronic pancreatitis.

The unbiased sequencing approach used in this study identified significantly differentially expressed microRNAs that are consistent with a potential mechanistic role in the pathogenesis of pancreatitis, as in humans.^27^, ^39^ The conserved miR‐221/222 cluster is an important regulator in multiple cellular processes, which is encoded tandemly in chromosome Xp11.3, and are highly homologous miRNAs sharing the same seed sequence and as a result were not differentiated by the Qiagen IPA analysis.^40^ A dog in this study had the highest levels of both miR‐211 and miR‐222, although high outliers of other microRNAs were from different dogs. Hsa‐miR‐221 is a distinct biomarker of human early chronic pancreatitis predicting early chronic pancreatitis with an area under the curve (AUC) of 100.0%.^29^ MiR‐221/222 are regulators of proliferation of pancreatic β‐cells and inhibit insulin production of pancreatic β‐cells in mice so their higher levels during pancreatic inflammation in these dogs would make biological sense.^41^ Qiagen IPA linked miR‐221/222 with inhibiting intracellular adhesion molecule‐1 (ICAM‐1). Pancreatic acinar cells upregulate the expression of ICAM‐1 as part of tumorigeneses to attract macrophages, so there could be a similar role for miR‐221/222 to translate ICAM‐1 during chronic pancreatic inflammation.^42^

MiR‐23a was higher in dogs with pancreatitis. MiR‐23a and miR‐23b concentrations are higher in human patients with severe acute pancreatitis (alongside hsa‐miR‐1260b, ‐762, ‐22‐3p).^43^ MiR‐23 promotes proliferation of pancreatic cancer cells and block apoptotic pathways.^44^ Qiagen IPA implicated an association with miR‐23a and the Smad family (Smad3 and Smad4). Activation of this pathway by miR‐23a and a role in pancreatic inflammation makes biological sense as Smad family proteins have a central role in pancreatic fibrosis by activating the proliferation of pancreatic stellate cells.^45^, ^46^, ^47^

MiR‐484 has utility as a biomarker for a number of diseases including pancreatic adenocarcinoma and putatively targets the *NOTCH3* gene in humans with chronic pancreatitis.^48^, ^49^ The Notch signaling pathway is active during pancreatic development and the reactivates during injury.^50^ *NOTCH3* expression is higher in the ducts of patients with chronic pancreatitis and is directly associated with fibrosis in other organs.^48^, ^51^, ^52^

MiR‐101‐3p was the only gene down‐regulated in this study. Qiagen IPA indicated a role of miR‐101 upregulating inducible co‐stimulator (ICOS) and prostaglandin‐2 (PTSG2). ICOS and PTGS2 are over‐expressed in the pancreas of humans with autoimmune pancreatitis and chronic pancreatitis, so this is also considered a relevant target to further examine in dogs with pancreatitis.^53^, ^54^, ^55^, ^56^

The limitations of this study include small sample groups, canine variability, lack of histopathology in all dogs and complicating concomitant disease in some of the dogs, which potentially influenced the pancreatitis scores assigned.^6^, ^36^, ^37^, ^57^ The McCord scale is validated for acute pancreatic disease, not the more chronic phenotype of the dogs in this study, so this could have also negatively influenced pancreatitis grade scoring. Canine pancreas‐specific lipase immunoreactivity was not measured in the control canine group, given the very poor sensitivity (21%) in dogs with mild pancreatitis.^14^, ^24^ Most of these limitations reflect the difficulties of researching pancreatitis in naturally occurring clinical disease.

## CONFLICT OF INTEREST DECLARATION

R.J. Mellanby is currently employed by IDEXX Laboratories Inc. and holds stock and stock options with IDEXX Laboratories, Inc. A. G. Gow currently works at Zoetis and holds stock and stock options with Zoetis. No other authors declare a conflict of interest.

## OFF‐LABEL ANTIMICROBIAL DECLARATION

The authors declare no off‐label use of antimicrobials.

## INSTITUTIONAL ANIMAL CARE AND USE COMMITTEE (IACUC) OR OTHER APPROVAL DECLARATION

Approved by the University of Edinburgh Veterinary Ethics Committee (approval number: 117.22).

## HUMAN ETHICS APPROVAL DECLARATION

Authors declare human ethics approval was not needed for this study.

## Supporting information


**Supplementary Figure 1.** Correlation between the microRNAs that were significantly differentially expressed in canine pancreatitis cases (n = 19).
