# Supplementary material for: Candidate circulating microRNA biomarkers in dogs with chronic pancreatitis
Source: J Vet Intern Med. 2024 Feb 13;38(2):995–1004. doi: 10.1111/jvim.17009 (PMC10937508; doi:10.1111/jvim.17009)
Supplement: Supplementary file 1 — Supplementary Figure 1. Correlation between the microRNAs that were significantly differentially expressed in canine pancreatitis cases (n = 19). [file JVIM-38-995-s001.pdf]

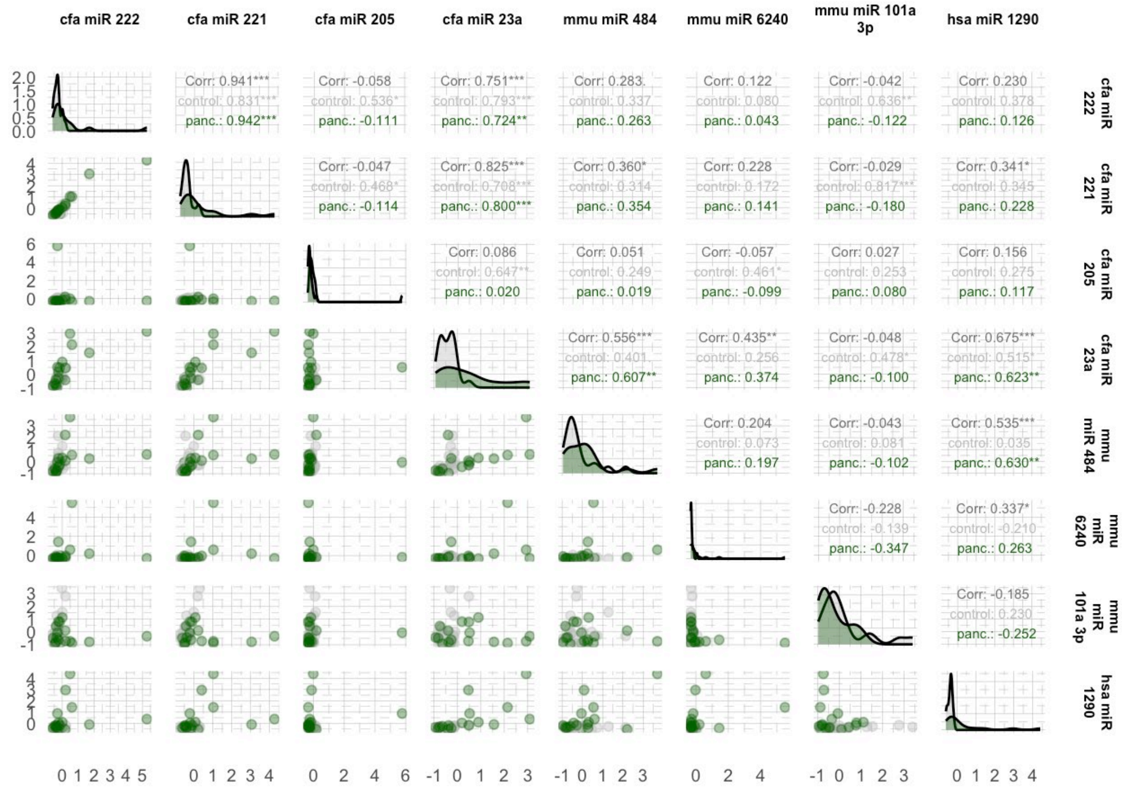

Supplementary Figure 1. Correlation between the microRNAs that were significantly differentially expressed in canine pancreatitis cases (n = 19). Plot generated using ggalley::ggpairs() function, in which Pearson correlation coefficients are calculated and significance thresholds reported as: *** p < 0.001, ** p < 0.01, * p < 0.05. Within 2 sets of microRNAs, expression was significantly correlated: miR-222, miR-221 and miR-23a; and miR-23a, miR-484 and miR-1290.
